# The Hammock Sign in Computed Tomography as a Detection Aid for Bicuspid Aortic Valves

**DOI:** 10.5334/jbsr.2974

**Published:** 2023-01-23

**Authors:** Daniel Devos, Charlotte Van Langenhove, Laurence Campens

**Affiliations:** 1 Ghent University Hospital, BE

**Keywords:** Aortic valve, Bicuspid Aortic Valve, Computed Tomography, Computed Tomographic Angiography

## Abstract

**Introduction:**

Bicuspid aortic valve is difficult to detect on standard transverse images.

**Purpose:**

We aimed to investigate the usefulness of the hammock sign for detection of bicuspid aortic valve.

**Methods:**

We retrospectively investigated the usefulness of a newly proposed ‘hammock sign’ in a population of 45 contrast enhanced computer tomographic studies to discern tricuspid (22) from anatomical bicuspid aortic (23) valves. The gold standard of aortic morphology was the definite diagnosis in the patient’s medical file, established by computed tomography, magnetic resonance, or surgery.

**Results:**

Computer tomographic (CT) studies of each aortic morphology were randomly evaluated for the presence of the hammock sign on coronal and sagittal images, by two readers blinded to the diagnosis. Sensitivity for detecting an anatomic bicuspid valve was 86%, and specificity was 100%.

**Conclusion:**

The hammock sign allows for a quick and easy diagnosis of an anatomical bicuspid aortic valve, merely by scrolling through the standard coronal reconstructions of any type of contrast-enhanced thoracic CT study, and regardless of any other findings associated with bicuspid aortic valve. Functional bicuspid aortic valves were not the scope of this study.

## Introduction

Bicuspid aortic valve (BAV) is the most common congenital heart defect in the general population, with a prevalence of 1–2% [1,2]. Bicuspid aortic valve has a particular association with ascending aortic dilatation, which is present in up to 80% of patients [3]. As dilatation progresses, it confers an increased risk for aortic dissection [4].

**Figure 1:**
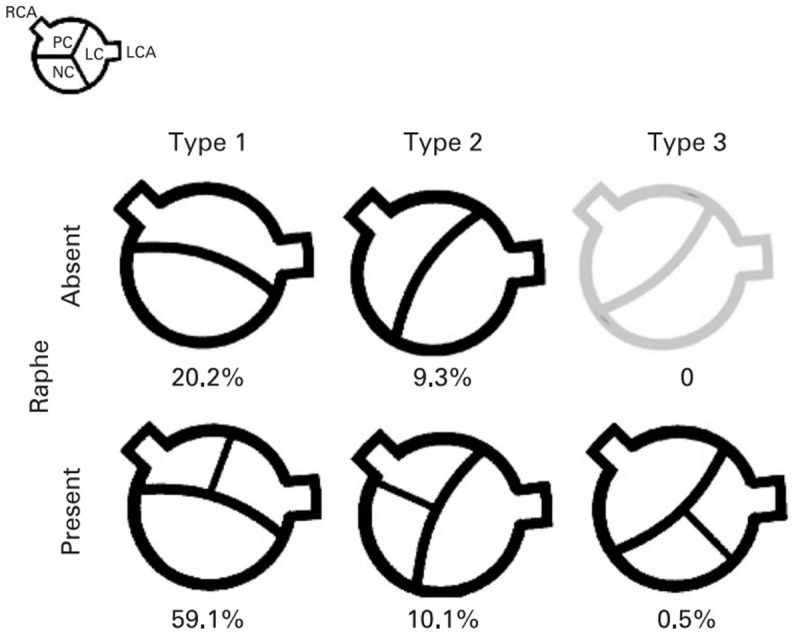
From Schaefer, et al [5]: orientation of real BAV (upper row, without a raphe), and functional BAV (lower row, with raphe).

A minority presents with an anatomical BAV (top row in Figure 1, from [5], i.e., two leaflets without a central commissure) [6].

Most BAV patients demonstrate a functional BAV i.e., two unequally sized leaflets, one of which has a central raphe that results from fusion of two leaflets (bottom row in Figure 1, from [5]. Functional BAV most often (>70% of cases) presents with fusion of right (RCC) and left (LCC) coronary cusp (anteroposterior (AP) orientation), whilst fusion of the RCC and non-coronary cusp (NCC) (laterolateral [LL] orientation) is seen in only 10–20%; fusion of the NCC and LCC is rare (5–10%). Routine ultrasound imaging substantially underestimates the prevalence and severity of BAV, and sensitivity of CT for BAV is only 67% [7].

In computed tomography (CT) imaging studies where the focus is not on the aortic valve, the diagnosis of bicuspid aortic valve is even more unlikely. This is mainly due to the double oblique orientation of the aortic valve, which largely varies between individuals. It is therefore difficult to spot the two or three cusps of the aortic valve while scrolling through a stack of transverse images. It is time consuming to reconstruct the double oblique image plane for optimal depiction of the aortic valve leaflets and cusps.

Delayed diagnosis of a bicuspid aortic valve conveys a risk for complications of associated aortic disease, of which ascending aortic aneurysm is the most common [3].

In this study we investigated the use of the new ‘hammock sign’ on coronal and sagittal contrast enhanced CT images for quick ‘scroll-by’ detection of a bicuspid aortic valve. Computed tomographic imaging data is, by default, presented in transverse images, but coronal and sagittal image planes are reconstructed easily and routinely.

**Figure 2:**
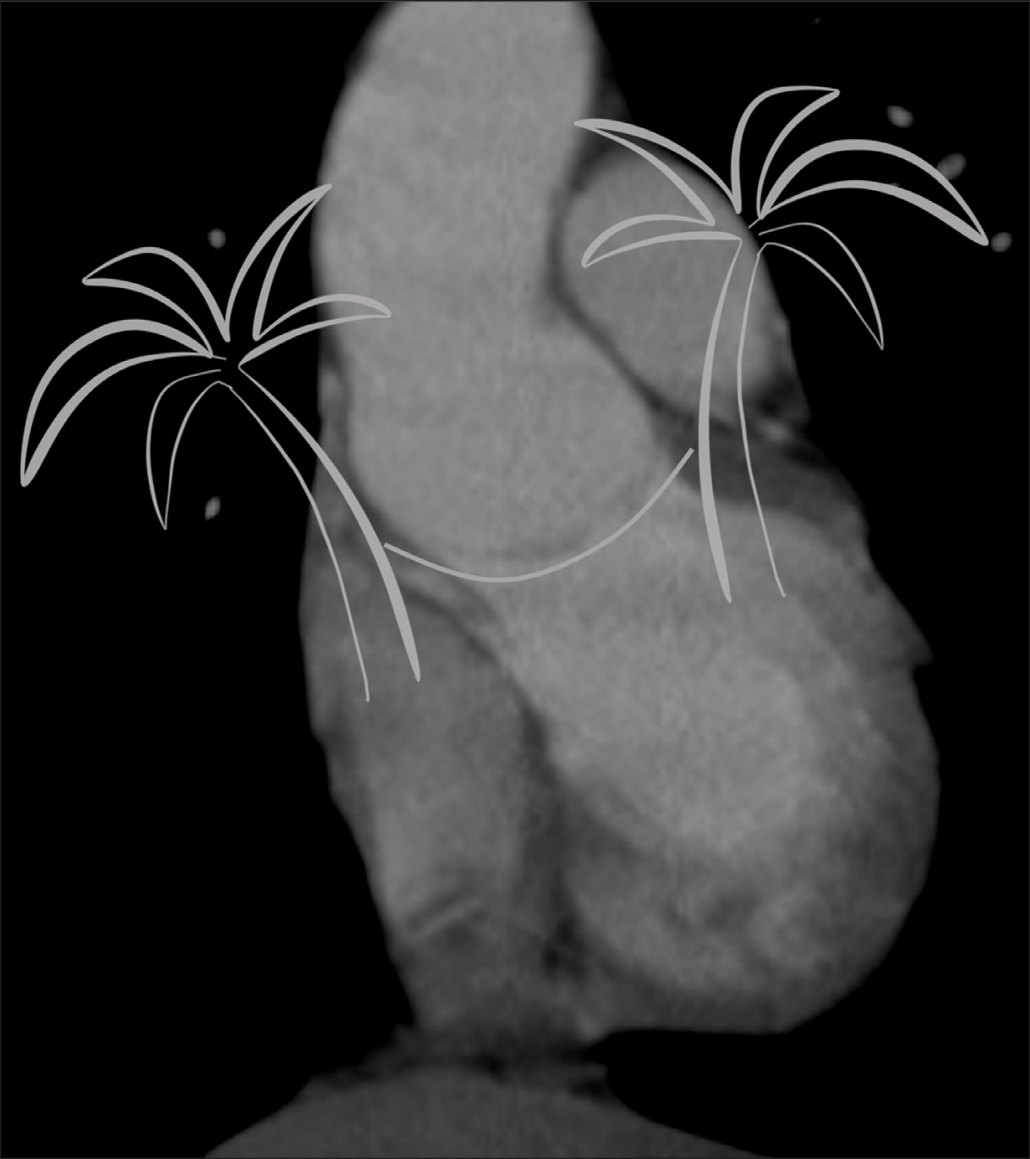
Bicuspid aortic valve leaflet spanning the width of the sinus in a curve resembling a hammock.

The hammock sign is inspired from the occasional depiction of a bicuspid aortic valve spanning the entire width of the aortic sinus on a coronal or sagittal reconstruction, its curve resembling a hammock (Figure 2).

## Materials and Methods

### Population

**Figure 3:**
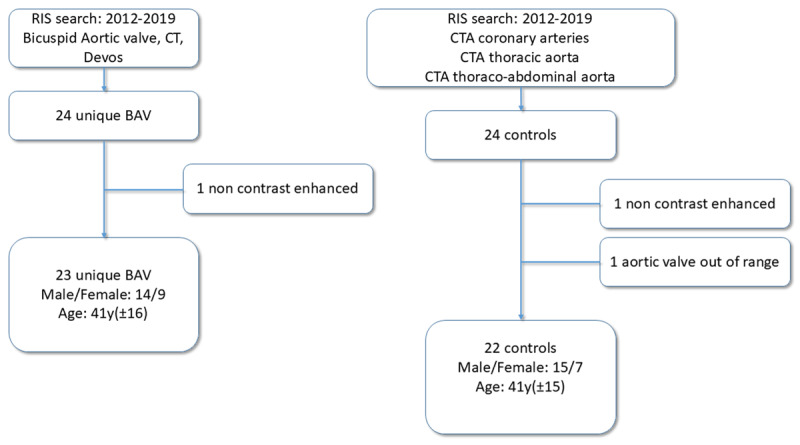
Flow diagram: Two RIS searches resulting in equally sized and nearly age-matched study groups.

The study was conducted retrospectively. Computed tomographic angiography (CTA) studies of coronary arteries and thoracic aorta were retrieved by the author not reviewing the images from the radiology information system (RIS), using the following cumulative criteria: reports dated between 2012–2019, written by a single cardiovascular radiologist (DD), and mentioning bicuspid aortic valve in either CTA or magnetic resonance angiography (MRA) of the same patient. As such, 24 CTA studies were retained (Figure 3). One non-contrast study was excluded.

### Control group

A control group was composed of 24 coronary and thoracic aortic CTA studies without the explicit mention of bicuspid aortic valve in the report. Studies without contrast agent and studies not including the aortic valve were excluded (Figure 3).

### Image evaluation

All studies, in random order and blinded from the report, were evaluated by an experienced radiologist (DD) for the presence of the ‘hammock sign’, using only the on the fly coronal and sagittal reconstructions of the thin slice stack: 0.6mm for cardiac studies, up to 2mm for CTA. The decision ‘bicuspid or tricuspid’ was supplemented by the subjective degree of certainty of the interpretation (maybe – probably – definitely). Three months later this evaluation was repeated by the first reader, blinded to the results of the first evaluation. All studies were also evaluated in the same fashion by a radiologist in training.

### Gold Standard

In a last round, all CTA exams were reconstructed in the aortic valve plane, to determine valve morphology; if unclear, another CTA or MRA study in the patient’s record was retrieved, or the medical record was consulted for the confirmation of the aortic valve’s morphology (eg. surgical record from ascending aortic replacement). Ultrasound findings were not taken into account.

Other descriptive parameters were noted: scan technique, use of Electrocardiogram (ECG) triggering, dilation of aortic root or ascending aorta, presence of coarctation.

Intra- and interobserver statistics were calculated using cross-tabulation and expressed with Cohen’s Kappa value.

This study was approved by our institution’s ethical review board. Individual informed consent was waived given the retrospective nature of the study and the number of dated CT investigations.

## Results

### Population

In this study population, 22 aortic valves were bicuspid, 21 were tricuspid, and only two were tricuspid with a bicuspid valve opening (functional BAV).

### Hammock sign and aortic valve

The presence of a hammock sign in both or one of the reconstruction planes resulted in the decision of a bicuspid aortic valve. This decision is cross-tabbed with the actual morphology of the valve as a gold standard (Table 1). Sensitivity for detecting an anatomic bicupid valve was 86%, and specificity 100%.

**Table 1:** Cross tabulation of BAV diagnosis based upon the hammock sign versus gold standard.


DECISION	GOLD STANDARD	

	BICUSPID VALVE	TRICUSPID VALVE/FUNCTIONAL BAV

Bicuspid	17	0

Tricuspid	4	23

Missing*	1	


(*) Missing: open valve: ‘probably bicuspid’ in first round.

**Figure 4:**
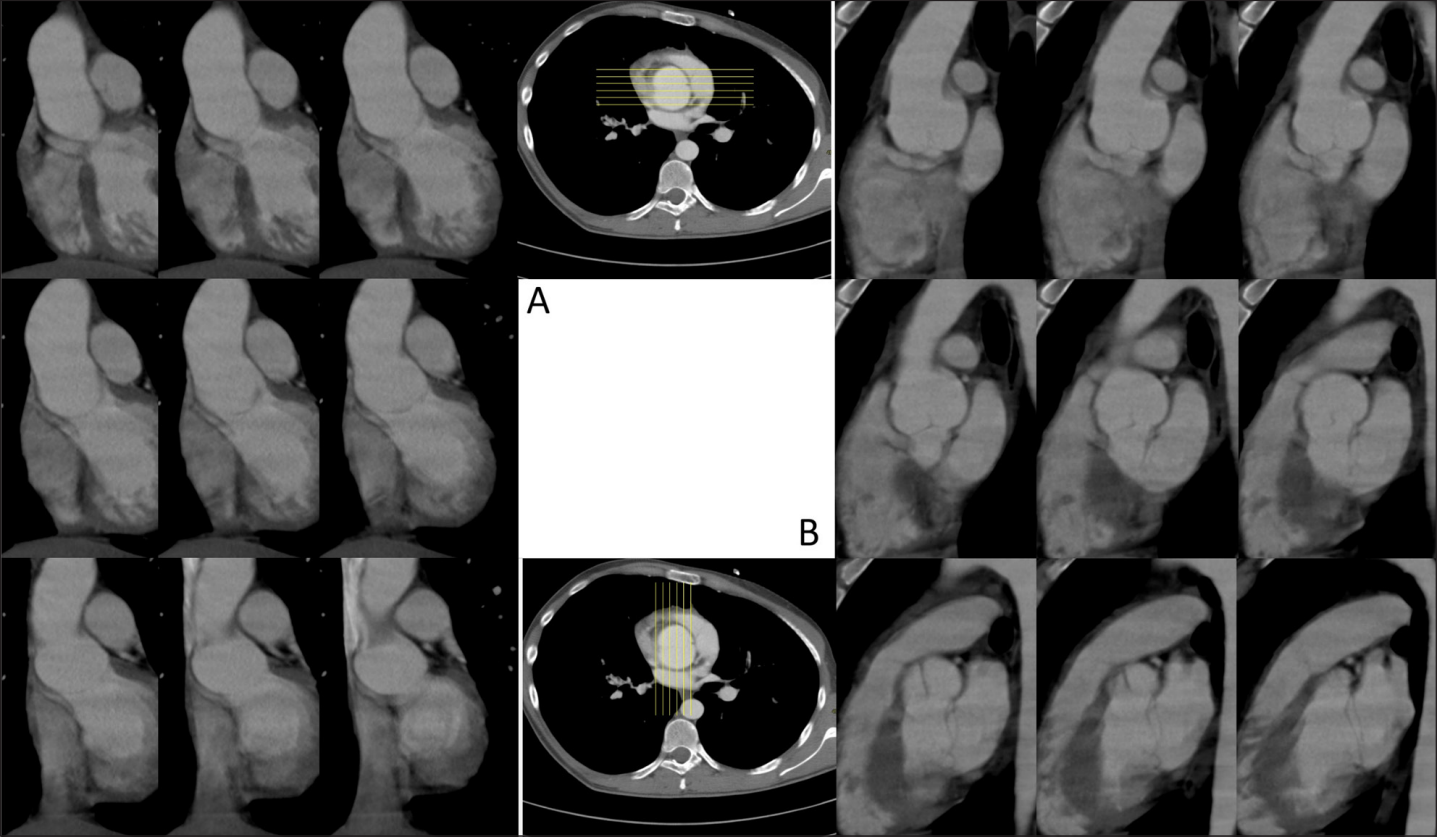
Upper Left (**A**): Scrolling through a coronal stack of images of a BAV which has a perfectly coronal coaptation plane. The hammock-like curve of the valve leaflets is present on every image: positive hammock sign. Lower Right (**B**): On the sagittal images there is no hammock-like curve at all.

**Figure 5:**
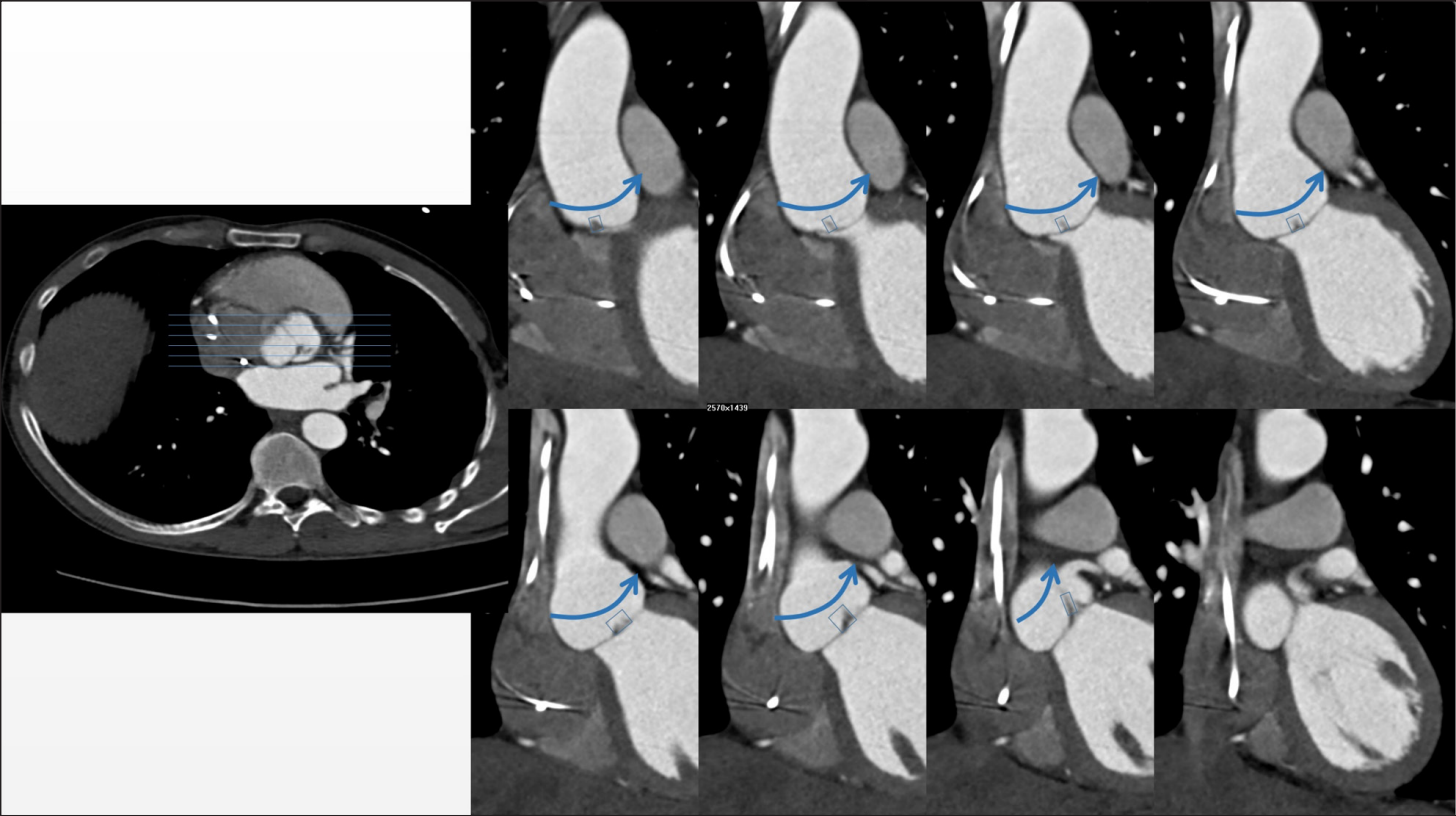
Scrolling through a coronal image stack of a BAV which has an oblique orientation to the coronal plane: the valve coaptation swings from (the patient’s) right to left.

**Figure 6:**
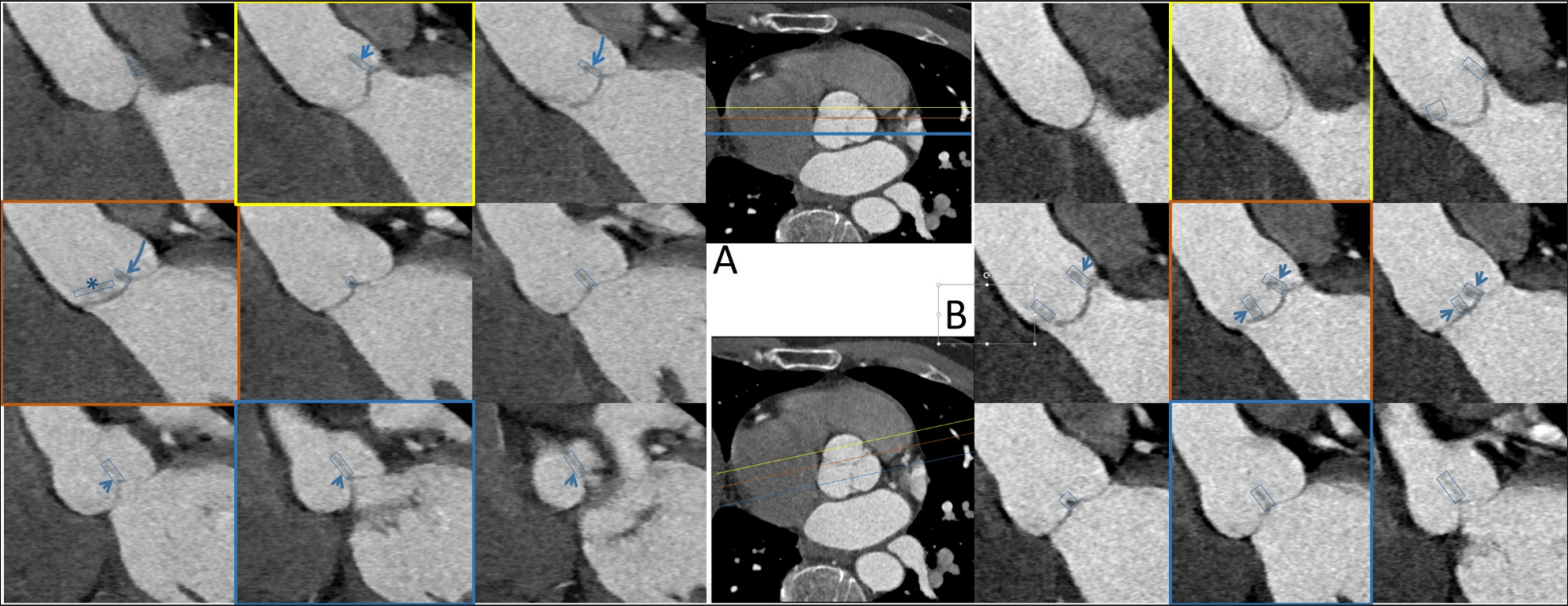
**A**. Scrolling through an oronal stack of images of a tricuspid aortic valve: the coaption plane of right and posterior leaflet has a coronal orientation and is thus rather inconspicuous (indicated with *). The two other coaptation planes can be seen moving inwards from the (patient’s) left, down to the middle of the sinus, and then up to the left again. In **B**, an oblique reconstruction to show images perpendicular to the left-and-posterior coaptation plane: two coaptation planes move inwards from either side, and the third remains centered.

When the hammock sign was called positive, it was visible on coronal images, and sometimes additionally on sagittal images too; it was never visible on sagittal images alone. When visible on both coronal and sagittal images, it was called a swinging hammock sign (Figs 4, 5 and 6, and their corresponding supplementary cine files).

Four BAV’s, without hammock sign on both coronal and sagittal image plane, were wrongly categorized as tricuspid (three times ‘probably’ and once ‘definitely’) by the first reader. The images were heavily motion artefacted.

The hammock sign was not seen on coronal or sagittal images in any tricuspid valve, including functional BAV’s.

### Computed Tomography

Fifteen CTA studies were performed with ECG triggering, displaying ten bicuspid and five tricuspid aortic valves. Different CT techniques were used: spiral CT, spiral CT with dual energy or dual source technique, and sequential ECG triggered.

Regardless of the valve morphology, the degree of certainty was nearly always ‘definitely’ when the scan technique included ECG triggering (13/15), and about half of cases (16/30) in non-ECG triggered technique (Table 2).

**Table 2:** Degree of certainty in ECG triggered and non-ECG-triggered series.


	ECG TRIGGERED	NO ECG

Definitely	13	16

Probably	2	12

Maybe	0	1

Missing	0	1


### Concomitant aortic changes

Twenty-five patients had a normal size of the aortic sinus and ascending aorta. Eight patients had mild aortic dilatation (sinus or ascending aorta), another eight showed moderate, and one severe dilatation. Three patients had had valve sparing ascending aortic replacement surgery.

### Reproducibility

The intra-observer variability was very good: Cohen’s Kappa = 0.9 (p << 0.01). Cross tabulation showed a moderate correlation between the two observers’ findings (Cohen’s kappa = 0.7 (p << 0.001)) (Table 3).

**Table 3:** Interobserver (a) and intra-observer (b) correspondence of BAV diagnosis based upon the hammock sign.


A.	DVD	

CVL	BICUSPID VALVE	TRICUSPID VALVE/SINUS

Bicuspid	18	2

Tricuspid	4	21

**B.**	**DVD2**	

**DVD1**	**BICUSPID VALVE**	**TRICUSPID VALVE/SINUS**

Bicuspid	18	1

Tricuspid	0	26


## Discussion

Our main findings were that the hammock sign was not seen on coronal or sagittal images in any tricuspid valve or functional BAV, and that a positive hammock sign was always present on coronal images, often on both coronal and sagittal images (swinging hammock), but not on sagittal images alone. Inter-observer variability was very good.

The authors propose a new sign for rapid coronal scroll-by detection of a BAV. It solely relates to valve morphology, independent of eventual concomitant aberrant aortic morphology.

On transverse images, it is often difficult to discriminate bicuspid from tricuspid valve. Indeed, the aortic valve has a double oblique orientation in reference to any orthogonal plane with much interindividual variation. However, with the use of thin slice image datasets, image reconstruction in coronal and sagittal plane has become standard in daily routine.

Figure 2 is taken from a coronal stack of images (as seen in Figure 4A) reconstructed from a non-ECG-triggered CTA study of a patient with a bicuspid aortic valve. This bicuspid valve is, coincidentally, perfectly aligned with the coronal plane. The sagittal image stack of the same study shows the coaptation of the leaflets on every image (Figure 4B). In any other orientation of the bicuspid aortic valve the coaptation of the leaflets will show to be swinging to and fro in the sinus when scrolling through the coronal and the sagittal images (Figure 5). We referred to this image as ‘swinging hammock’ sign.

For comparison, coronal reconstruction of a tricuspid aortic valve is shown in Figure 6A. When scrolling through the image set, one coaptation of the leaflets moves to the middle of the sinus, one coaptation lies inconspicuously in the valve plane (*) and the third coaptation returns from the middle of the sinus to the same side of the aortic sinus.

The same dataset was reconstructed obliquely, to show the situation where one of the three coaptation segments is perpendicular to the image reconstruction plane (Figure 6B).

Our aortic hammock sign should not be confused with the hammock sign of the coronary artery that has been proposed for the description of the exceptional course of the left main coronary artery dipping downwards into the interventricular septum and then sloping upwards before it bifurcates into left anterior descending and circumflex arteries [8].

The hammock mitral valve denotes a congenital abnormal morphology, and it is as such not an imaging sign [9].

This study aims to evaluate the aortic hammock sign on sagittal and coronal images combined in any contrast enhanced thoracic CT study, with or without ECG-triggering, in arterial or venous phase scans.

The hammock sign is evaluated at a glance, by quickly scrolling through the aortic valve, mainly in the coronal plane, maybe also in the sagittal plane.

The hammock sign is a dynamic sign, i.e., it can only be seen by scrolling through a stack of images. Indeed, in a tricuspid aortic valve, a single image may be found where one leaflet is depicted at its largest (see Figure 4B, upper row). The hammock sign can only be called positive if this pattern is present on all images across the aortic sinus. One cannot evaluate the hammock sign on a still image.

Finding a hammock sign on coronal or sagittal image stack, or both, implies that the aortic valve is bicuspid and should be further examined, if possible with a reconstruction in the aortic valve plane. In our population, all the hammock sign positive image datasets were subsequently confirmed to represent bicuspid aortic valves.

The absence of the hammock sign does not exempt the CTA reader from adequate reconstruction of the aortic valve plane image, especially if a possible bicuspid valve morphology was the reason for the CTA referral.

Whether the hammock sign is present on coronal or on sagittal images depends on the orientation of the aortic root, and the phenotype of the bicuspid valve. Aortic bicuspidy can result from the fusion of left and right cusp (73%), of right and posterior (non-coronary) cusp (24%), or of left and posterior cusp (3%) [10].

In our small population, there was no combination of a hammock sign presenting on sagittal images only, and not on coronal images, which seems consistent with the presentation of bicuspid valves without raphe according to Figure 1, from Schaefer et al [5], it would require an unusual rotation of a type 2 valve without raphe to show up as a hammock on sagittal images only. Still, it remains useful to inspect the aortic valve on sagittal images, to add to the confidence of the diagnosis, since a coaptation segment of a tricuspid valve lying in plane with the reconstructed image can be very inconspicuous.

Findings on ECG triggered CT studies provide far more certainty as to the aortic morphology. However, ECG triggered studies are nearly exclusively performed in the setting of a cardiologic clinical query, where the radiologist will indeed focus his attention on the heart and its valves. This report on the use of the hammock sign is aimed at a widespread application in any contrast enhanced CT study. The hammock sign represents bicuspidy on any contrast enhanced CT image, even though it may manifest far less clearly in venous phase.

We did not evaluate non-contrast images, as the aortic leaflets cannot be distinguished. Leaflet calcification will most probably impede recognition of the hammock sign.

The hammock sign cannot be seen when the aortic valve is open (scanned in systole). One CT study, scanned without ECG triggering, showed an open aortic valve: hammock sign was therefore absent (evaluated as ‘undecided’, ‘missing’ in statistics).

The spectrum of aortic bicuspidy was reflected in our results by the presence of two tricuspid sinuses with a bicuspid valve opening. Most probably, the hammock sign fails to detect those functional BAV’s as the method relies on valve leaflet morphology in the closed state and not on valve opening morphology. At three evaluations each, both functional BAV were categorized as ‘definitely tricuspid’ three times, ‘probably tricuspid’ twice, and ‘maybe bicuspid’ just once.

Other findings associated with bicuspid aortic valve are dilatation of the aortic root or ascending aorta, and coarctation. In the presence of these abnormalities, reconstruction of a double oblique aortic valve plane is mandatory.

The strengths of this study are the selection of the study population, the integrity of the gold standard, and the inter- and intra-observer evaluation of the sign. The sign is new and easily applicable for any radiologist in the clinical routine of any contrast enhanced thoracic CT study. Hardly any learning curve is needed to start looking for this sign. Evaluating the aorta for this sign on coronal and/or sagittal images requires no special software and only the time of a quick focused look. The sign is directly and exclusively related to the valve, independent of concomitant aortic pathology.

This study has a few limitations: there is but a small number of subjects. Especially the number of functional BAV, not being the focus of this study, is too low to determine the detection rate of functional BAV. This sign would probably not perform well in the larger part of the spectrum of BAV disease. In a static imaging method such as CT detection of a raphe in an aortic valve, even with dedicated valve plane reconstruction, will always be challenging. With this new sign, the authors hope to raise attention to the aortic valve in a way that is accessible for all radiologists. We hope that this sign be used as a fast tool for screening for aortic valve pathology.

## Conclusion

The novel hammock sign is a finding that can lead to the incidental ‘scroll-by’ diagnosis of an anatomical bicuspid aortic valve in any contrast enhanced CT study, independently of dilated ascending aorta and coarctation. If positive, confirm bicuspidy with double oblique aortic valve plane reconstruction. If not present, there is only a very small risk of missing an anatomical bicuspid aortic valve. The hammock sign is not validated in a larger population of functional bicuspid valve.

## Summary Statement

We propose the new ‘hammock sign’ for quick scroll-by detection of anatomical bicuspid aortic valve on any contrast enhanced thoracic CT study.

## Key Results

1. Hammock sign is for quick scroll-by detection of bicuspid aortic valve on tomography.
2. Hammock sign on standard coronal images represents definitive diagnosis of anatomical bicuspid aortic valve.
3. Absence of hammock sign on standard coronal images renders the diagnosis of an anatomical bicuspid aortic valve very unlikely.

## Abbreviations

AP: Antero-Posterior

BAV: Bicuspid Aortic Valve

CTA: Computer Tomographic Angiography

ECG: Electrocardiogram

LCC: Left Coronary Cusp

LL: Latero-Lateral

MRA: Magnetic Resonance Angiography

NCC: Non-Coronary Cusp

RCC: Right Coronary Cusp

RIS: Radiology Information System
